# Paraneoplastic neurological syndrome and its impact on the treatment outcomes of small‐cell lung cancer: A single‐center retrospective analysis

**DOI:** 10.1111/1759-7714.15472

**Published:** 2024-10-19

**Authors:** Yuki Sato, Satoru Fujiwara, Chigusa Shirakawa, Ryosuke Hirabayashi, Kazuma Nagata, Atsushi Nakagawa, Ryo Tachikawa, Keisuke Tomii

**Affiliations:** ^1^ Department of Respiratory Medicine Kobe City Medical Center General Hospital Kobe Japan; ^2^ Department of Neurology Kobe City Medical Center General Hospital Kobe Japan

**Keywords:** anticancer therapy, paraneoplastic neurological syndrome, performance status, small‐cell lung cancer

## Abstract

**Introduction:**

Paraneoplastic neurological syndrome (PNS) is associated with small‐cell lung cancer (SCLC). However, the frequency and characteristics of PNS and the efficacy of anticancer treatment for these patients have not been investigated in the Japanese/Asian population previously. Therefore, we aimed to better understand PNS by evaluating real‐world data from patients with PNS complicated by SCLC.

**Methods:**

Patients diagnosed with Stage II–IV SCLC at a single center between August 2007 and April 2021 were retrospectively analyzed. The primary outcome was the incidence of PNS. The secondary outcomes were the change in performance status (PS) after treatment commencement and outcomes following anticancer treatment, including objective response rate (ORR), progression‐free survival (PFS), and overall survival (OS).

**Results:**

A total of 318 patients were evaluated; PNS was present in 2.8% (*n* = 9) of the overall population. All patients with PNS exhibited poor Eastern Cooperative Oncology Group PS (≥2); moreover, 78% of patients had a PS score of 3–4. An improvement in PS was observed in 56% (*n* = 5) of patients. Patients with PNS exhibited treatment efficacies similar to patients without PNS (ORR: 89% vs. 83%, *p* = 1.0; PFS: 7.6 vs. 5.7 months, *p* = 0.69; OS: not reached vs. 15.6 months, *p* = 0.23).

**Conclusions:**

A total of 2.8% of patients had SCLC complicated by PNS, with poor PS observed. However, anticancer therapy led to an improvement in PS and comparable ORR, as well as PFS and OS similar to those observed in patients without PNS. Thus, anticancer therapy should be considered in patients with PNS.

## Graphical abstract

Patients diagnosed with small‐cell lung cancer (SCLC) at a single center between August 2007 and April 2021 were retrospectively analyzed. A total of 318 patients were evaluated; paraneoplastic neurological syndrome (PNS) was present in 2.8% (*n* = 9) of the overall population. All patients with PNS exhibited poor Eastern Cooperative Oncology Group PS (≥2). An improvement in performance status was observed in 56% (*n* = 5) of patients. Patients with PNS exhibited treatment efficacies similar to patients without PNS (PFS: 7.6 vs. 5.7 months, *p* = 0.69). Anticancer therapy should be considered in patients with PNS.

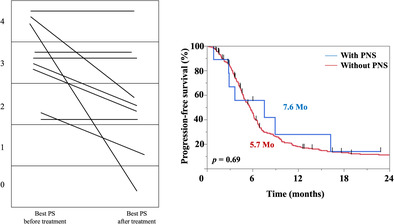

## INTRODUCTION

Lung cancer is the leading cause of cancer‐related mortality worldwide, with small‐cell lung cancer (SCLC) accounting for 15%–20% of all lung cancer cases.^1^ SCLC is notorious for its early metastatic spread and its initial but transient sensitivity to chemotherapy.

Paraneoplastic neurological syndrome (PNS) is a rare autoimmune disorder that has been recognized as a devastating comorbidity. PNS results from the indirect effect of a tumor on the nervous system without local invasion or metastasis and occurs in approximately 0.1% of patients with cancer.^2^ PNS has diverse presentations, ranging from being a relatively isolated syndrome (e.g., cerebellar degeneration) to a generalized syndrome (e.g., encephalomyelitis). Previous reports have indicated that half of PNS cases are associated with SCLC^3^ and that the prevalence of PNS in patients with SCLC is estimated to be 2%–9.4%.^4^, ^5^ Autoantibodies play an important role in PNS and are found in 31%–57% of patients with SCLC complicated by PNS.^6^, ^7^ Taken together, these findings suggest that patients with PNS exhibit distinctive features.

Currently, there exists no standard of care or firm evidence for patients with PNS. When oncologists encounter patients with SCLC accompanied by PNS, the management strategy becomes difficult owing to worsening neurological symptoms and a lack of tolerability for the initiation of anticancer therapy. Steroids or immunomodulatory therapies have been reported to exhibit a relatively limited effectiveness in controlling neurological diseases.^8^ In this setting, from the viewpoint of pathogenesis, tumor control is critical for these patients.^9^, ^10^ Generally speaking, anticancer therapies are recommended for patients with poor performance status (PS), in case the impairment in PS originates from the tumor burden.^11^ For example, tumor removal for anti‐*N*‐methyl‐d‐aspartic acid receptor encephalitis has been reported to be associated with an improvement in outcomes and a better treatment response.^12^ Nonetheless, comprehensive clinical data on anticancer therapies for patients with PNS are currently lacking. As the number of patients with PNS is highly limited, randomized trials cannot therefore be conducted. Moreover, current evidence obtained from published case reports is highly biased toward interpretations of treatment benefits.^13^ Hence, data obtained from the real‐world setting are needed.

In the present study, we aimed to reveal the precise frequency and characteristics of PNS in the Japanese population, as well as the efficacy of anticancer therapy for PNS, by systematically analyzing an unselected and unbiased SCLC patient cohort from a single institution with a long follow‐up period. We believe that this study would provide a better understanding of PNS and its treatment.

## MATERIALS AND METHODS

### Study population

**FIGURE 1:**
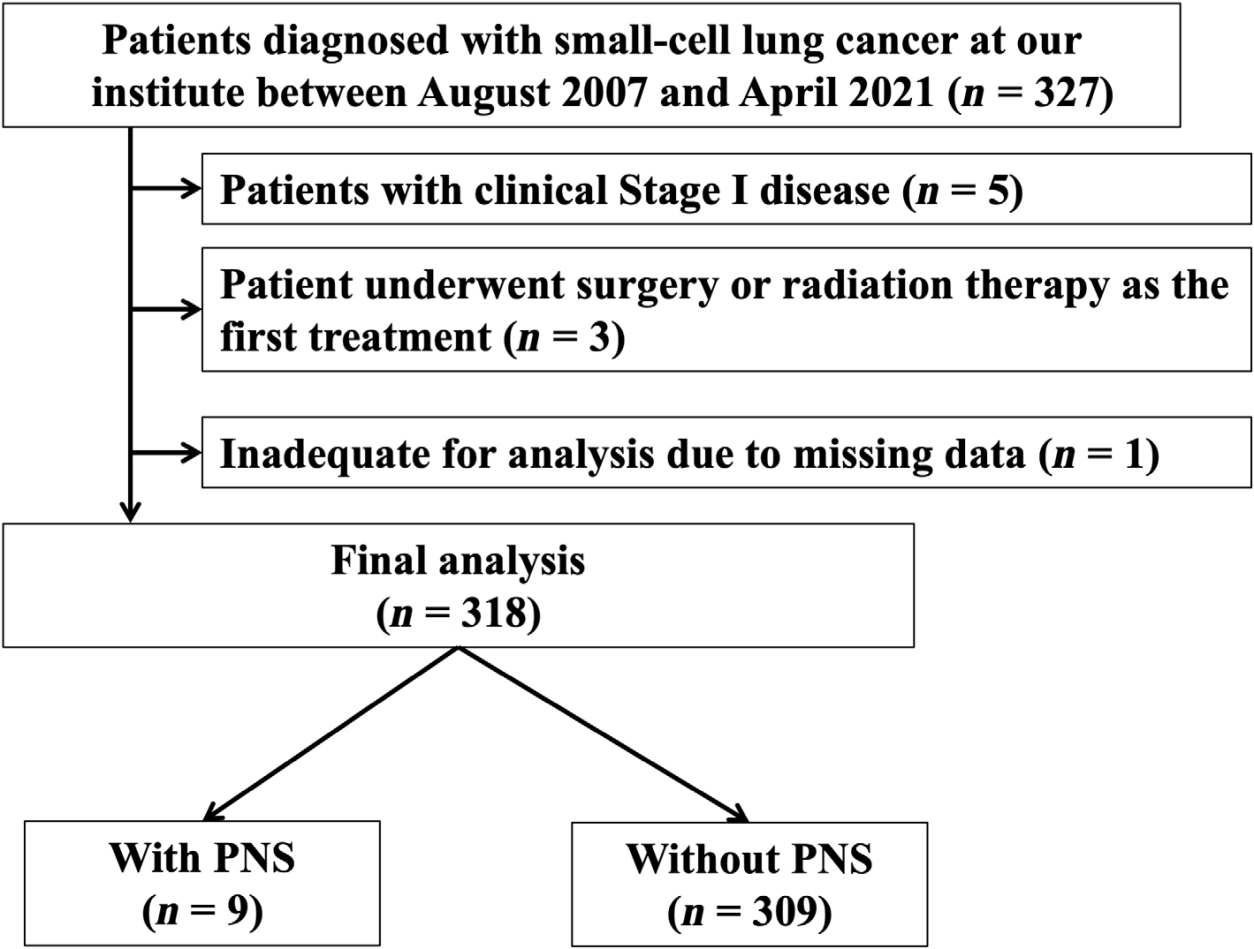
Flowchart of this study. PNS, paraneoplastic neurological syndrome.

Patients diagnosed with SCLC at Kobe City Medical Center General Hospital (Kobe, Japan) between August 2007 and April 2021 were retrospectively collected from the admission database. Figure 1 shows the patient flowchart for this study. We focused on patients with locally advanced or advanced disease (Stages II–IV) who were candidates for chemotherapy or chemoradiotherapy. Patients with stage I disease (*n* = 5) were excluded. Patients who only underwent surgery or radiation therapy for SCLC were also excluded (*n* = 3), as these treatments were not recognized as the standard of care. This cohort included all patients diagnosed with locally advanced or advanced SCLC at our institute during the indicated period.

SCLC was diagnosed by pathologists according to the third or fourth World Health Organization classification of lung cancer.^14^, ^15^ If the diagnosis was difficult based solely on hematoxylin and eosin–stained sections, neuroendocrine immunohistochemistry results were evaluated as appropriate. The following data were collected from electronic medical records: age, sex, Eastern Cooperative Oncology Group (ECOG) PS at diagnosis, smoking status, ethnicity, clinical stage, SCLC subtype (pure SCLC or combined), comorbidities, diagnosis of other cancers, location of the primary lesion, and site of metastasis. The data cutoff date was set to September 31, 2021. Older patients were defined as those aged ≥75 years, and poor PS was defined as an ECOG PS score of ≥2. Progression‐free survival (PFS) was defined as the time from receiving the first dose of chemotherapy to lung cancer progression, death from any cause, or end of follow‐up. Overall survival (OS) was defined as the time from receiving the first chemotherapy dose to death. All patients were classified according to clinical stage based on the eighth edition of the TNM classification system and were subsequently categorized into extensive disease or limited disease.^16^ Clinical tumor assessment was performed using the Response Evaluation Criteria in Solid Tumors version 1.1. Some patients were evaluated by the Response Evaluation Criteria in Solid Tumors version 1.0 in the inclusion period. They were re‐evaluated by reviewing the radiographic records.

This study was approved by our institutional ethics committee (approval no. zn230614) and was conducted in accordance with the principles embodied in the 1964 Declaration of Helsinki and its later amendments. Informed consent was obtained from patients in written form or through an opt‐out approach on the website. The study protocol was designed according to the Strengthening the Reporting of Observational Studies in Epidemiology guidelines.

### PNS evaluation

When patients were suspected of having PNS, a neurologist from our team was consulted and performed full neurological examinations. Additionally, serum samples were evaluated for autoantibodies, and neurological physicians conducted electroencephalography and nerve conduction tests, as appropriate. The presence of PNS was evaluated based on the diagnostic criteria published by Gaus in 2004, and the diagnosis was reconfirmed using the criteria published by Gaus in 2021.^17^ In case of definite or probable diagnosis based on the 2021 criteria, the patients were considered to have PNS. Follow‐up clinical data were obtained from medical records for all patients.

### Study design

As this study employed a retrospective design, the sample size was neither pre‐specified nor calculated. The primary outcome was the incidence of PNS after SCLC diagnosis. The secondary outcomes were the change in the ECOG PS after the commencement of anticancer treatment and the treatment outcomes, including the objective response rate (ORR), PFS, and OS.

## STATISTICAL ANALYSIS

Continuous variables are expressed as medians and interquartile ranges (IQRs), whereas categorical data are presented as absolute values and percentages. Continuous variables were analyzed using the Student's *t*‐test. Dichotomous variables were examined using Fisher's exact test. The Cox proportional hazards model was used to estimate the hazard ratios for factors associated with survival in both univariate and multivariate analyses. Multivariate analysis was conducted on all important clinical factors (age, ECOG PS, presence of interstitial lung disease, and extensive disease or limited disease, and use of chemotherapy, radiation therapy, or immune checkpoint inhibitors [ICIs]). PFS and OS were estimated using the Kaplan–Meier method, and the groups were compared using the log‐rank test. Statistical significance was set at a two‐tailed *p*‐value of <0.05. Statistical analyses were performed using the JMP 16 software (SAS Institute, Cary, NC, USA).

## RESULTS

### Patient characteristics

Table 1 summarizes the patient characteristics. A total of 318 patients were evaluated; of these patients, 9 had PNS (Figure 1). PNS was present in 2.8% of the overall population. The median patient age was 72.0 years (IQR: 66–77 years), and 80% (*n* = 255) of patients were male, 33% (*n* = 104) had limited disease, and 96% (*n* = 306) were current/former smokers. According to TNM staging, 0.3% (*n* = 1) had Stage IIA, 4% (*n* = 12) had Stage IIB, 18% (*n* = 56) had Stage IIIA, 10% (*n* = 31) had Stage IIIB, 5% (*n* = 16) had Stage IIIC, 23% (*n* = 74) had Stage IVA, and 40% (*n* = 128) had Stage IVB disease. Most patients were of Japanese descent. Combined SCLC was found in 11% (*n* = 36) of patients. As for metastatic sites, 16% (*n* = 51) of patients showed brain metastasis. Second primary cancer other than SCLC was found in 20% (*n* = 63) of patients. In terms of comorbidity, interstitial lung disease was found in 23% (*n* = 73) of patients. In terms of treatment modality, 69% (*n* = 219), 4% (*n* = 14), 22% (*n* = 70), and 5% (*n* = 15) of patients received chemotherapy alone, chemotherapy and ICI therapy, chemoradiation therapy, and best supportive care, respectively. The median follow‐up period was 12.4 months (IQR: 6.2–22.6). The median OS was 15.6 months, whereas the median PFS was 5.7 months; the overall treatment response rate was 84% (Figure S1 and Table S1). Patient baseline characteristics did not differ significantly between patients with PNS and those without PNS.

**TABLE 1:** Characteristics of patients with and without PNS.

	Total (*N* = 318)	Patients with PNS (*N* = 9)	Patients without PNS (*N* = 309)	*p*‐value
Age (years), *n* (%)				
<75	205 (64)	7 (78)	198 (64)	0.50
≥75	113 (36)	2 (22)	111 (36)	
Mean (SD)	71.2 (8.5)	71.4 (8.5)	66.6 (7.8)	0.09
Sex, *n* (%)				
Male	255 (80)	8 (89)	247 (80)	1.0
Female	63 (20)	1 (11)	62 (20)	
Ethnicity, *n* (%)				
Japanese	316 (99)	9 (0)	307 (99)	1.0**
Chinese	1 (0.3)	0 (0)	1 (0.3)	
Indian	1 (0.3)	0 (0)	1 (0.3)	
ECOG PS, *n* (%)				
0–1	217 (68)	0 (0)	217 (70)	<0.001
2–4	101 (32)	9 (100)	92 (30)	
Smoking status, *n* (%)				
Current or former	306 (96)	9 (100)	297 (96)	1.0*
Never	10 (3)	0 (0)	10 (3)	
Missing data	2 (1)	0 (0)	2 (1)	
Stage, *n* (%)				
IIA	1 (0.3)	0 (0)	1 (0.3)	
IIB	12 (4)	0 (0)	12 (4)	
IIIA	56 (18)	3 (33)	53 (17)	
IIIB	31 (10)	0 (0)	31 (10)	
IIIC	16 (5)	0 (0)	16 (5)	
IVA	74 (23)	3 (33)	71 (23)	
IVB	128 (40)	3 (33)	125 (40)	
LD or ED, *n* (%)				
LD	104 (33)	3 (33)	101 (33)	1.0
ED	214 (67)	6 (67)	208 (67)	
Primary site, *n* (%)				
Right upper lobe	102 (32)	1 (11)	101 (33)	
Right middle lobe	18 (6)	0 (0)	18 (6)	
Right lower lobe	57 (18)	2 (22)	55 (18)	
Left upper lobe	70 (22)	3 (33)	67 (22)	
Left lower lobe	49 (15)	0 (0)	49 (16)	
Central type	22 (7)	3 (33)	19 (6)	
Combined case, *n* (%)				
Yes	36 (11)	0 (0)	36 (12)	0.6
No	282 (89)	9 (100)	273 (88)	
Metastasis site, *n* (%)				
Brain	51 (16)	0 (0)	51 (17)	
Lung	22 (7)	0 (0)	22 (7)	
Bone	68 (21)	0 (0)	68 (22)	
Liver	77 (24)	3 (33)	74 (24)	
Adrenal	27 (8)	2 (22)	25 (8)	
Malignant effusion/pleural	63 (20)	0 (0)	63 (20)	
Other	39 (12)	3 (33)	36 (12)	
Second primary cancer, *n* (%)				
Yes	63 (20)	1 (11)	62 (20)	0.69
No	255 (80)	8 (99)	247 (80)	
Interstitial lung disease, *n* (%)				
Present	73 (23)	0 (0)	73 (24)	0.15
Absent	245 (77)	9 (100)	236 (76)	
Other comorbidities, *n* (%)				
Angina pectoris/myocardial infarction	28 (9)	0 (0)	28 (9)	
Chronic heart failure	10 (3)	0 (0)	10 (3)	
Peripheral vascular disease	7 (2)	0 (0)	7 (2)	
Dementia	10 (3)	0 (0)	10 (3)	
Cerebral infarction	22 (7)	0 (0)	22 (7)	
COPD	27 (9)	0 (0)	27 (9)	
Autoimmune	22 (7)	1 (11)	21 (7)	
Gastric ulcer	18 (6)	2 (22)	16 (5)	
Liver	14 (4)	1 (11)	13 (4)	
Diabetes mellitus	79 (25)	2 (22)	77 (25)	
Chronic renal failure	13 (4)	0 (0)	13 (4)	
Treatment				
Chemotherapy	219 (69)	9 (100)	210 (68)	
Chemotherapy and ICI	14 (4)	0 (0)	14 (5)	
Chemoradiotherapy	70 (22)	0 (0)	70 (23)	
Best supportive care	15 (5)	0 (0)	15 (5)	

Abbreviations: ECOG PS, Eastern Cooperative Oncology Group Performance Status; ED, extended disease; ICI, immune checkpoint inhibitor; IQR, interquartile range; LD, limited disease; PNS, paraneoplastic neurological syndrome.

* Comparison between never and current/former smokers.

** Comparison between Japanese and non‐Japanese.

Table 2 presents the profile details of patients with PNS. According to the PNS diagnostic criteria, there were six cases of definite diagnosis and three of probable diagnosis. Among patients with PNS, Lambert–Eaton myasthenic syndrome was found in four patients, encephalomyelitis in two patients, and limbic encephalitis, cerebellar degeneration, and peripheral neuropathy in one patient each. Among these patients, we found three patients with P/Q‐type Ca channel antibody and one patient with N‐type Ca channel antibody, Recoverin, PCA‐2, and Hu antibody. All patients with PNS exhibited poor ECOG PS (≥2); moreover, 78% of patients had a PS score of 3–4. Patients with PNS and those without PNS did not differ with respect to other clinical backgrounds. One‐third of patients with PNS had limited disease. In all patients, the decline in PS was attributed to PNS. Eight of nine patients developed manifestations of PNS before the diagnosis of lung cancer. All patients with PNS were started on chemotherapy without ICI therapy, including general intensive care such as endotracheal intubation and intravenous high‐dose immunoglobulin therapy. Most attending physicians reduced the chemotherapy dose from the standard dose. The dose reduction rate was 33%, and the chemotherapy completion rate was 67%.

**TABLE 2:** Characteristics of patients with PNS.

Patient no.	Age	Sex	ED or LD	PNS type/diagnostic level	Antibodies	Anticancer treatment/dose reduction/completion	Other therapies	ORR	ECOG PS change	PFS (months)	OS (months) (cause of death)	Cause of PS deterioration	Timing of PNS manifestation
1	56	Male	ED	Lambert–Eaton myasthenic syndrome/definite	P/Q‐type Ca channel Ab	Cb^5^/VP(80) No Yes	IVIG Steroid Diaminopyridine	PR	2 to 1	6.0	6.0 (censored)	PNS	Before diagnosis
2	70	Male	LD	Lambert–Eaton myasthenic syndrome/definite	P/Q‐type Ca channel Ab N‐type Ca channel Ab	Cb^5^/VP(80) RT (definite) No Yes	IVIG Diaminopyridine	PR	4 to 1	22.8	22.8 (censored)	PNS	Before diagnosis
3	71	Male	ED	Cerebellar degeneration/probable	Recoverin	Cb^5^/VP(80) Yes No	IVIG	PR	3 to 3	2.9	2.9 (censored)	PNS	Before diagnosis
4	66	Male	LD	Limbic encephalitis/definite	PCA‐2	Cb^5^/VP(80) Yes No	IVIG Steroid	PR	3 to 2	16.3	19.3 (censored)	PNS	Before diagnosis
5	77	Male	LD	Lambert–Eaton myasthenic syndrome/definite	P/Q type Ca channel Ab	Cb^5^/VP(80) No Yes	IVIG Steroid	PR	4 to 2	2.9	5.0 (Lung cancer progression)	PNS	Before diagnosis
6	57	Male	ED	Peripheral neuropathy/definite	Hu	Cb^5^/VP(80) No Yes	IVIG Steroid	PR	2 to 2	3.7	22.3 (Lung cancer progression)	PNS and lung cancer tumor burden	After diagnosis
7	76	Female	ED	Encephalomyelitis/probable	None	VP (100) + RT (palliative) Yes Yes	IVIG Plasma exchange	PD	3 to 2	7.6	8.8 (censored)	PNS	Before diagnosis
8	67	Male	ED	Encephalomyelitis/probable	None	Cb^2^/CPT‐11(40) No No	Invasive ventilation	PR	4 to 4	0.9	2.0 (PNS progression)	PNS	Before diagnosis
9	59	Male	ED	Lambert–Eaton myasthenic syndrome/definite	P/Q type Ca channel Ab	CDDP (60)/CPT‐11(60) No Yes	None	PR	3 to 3	9.0	11.6 (censored)	PNS	Before diagnosis

Abbreviations: Cb, carboplatin; CPT‐11, irinotecan; ECOG PS, Eastern Cooperative Oncology Group Performance Status; ED, extended disease; IVIG, intravenous high‐dose immunoglobulin therapy; LD, limited disease; ORR, objective response rate; OS, overall survival; PD, progressive disease; PR, partial response; PFS, progression‐free survival; PNS, paraneoplastic neurological syndrome; VP, etoposide.

### Changes in the ECOG PS before and after treatment

**FIGURE 2:**
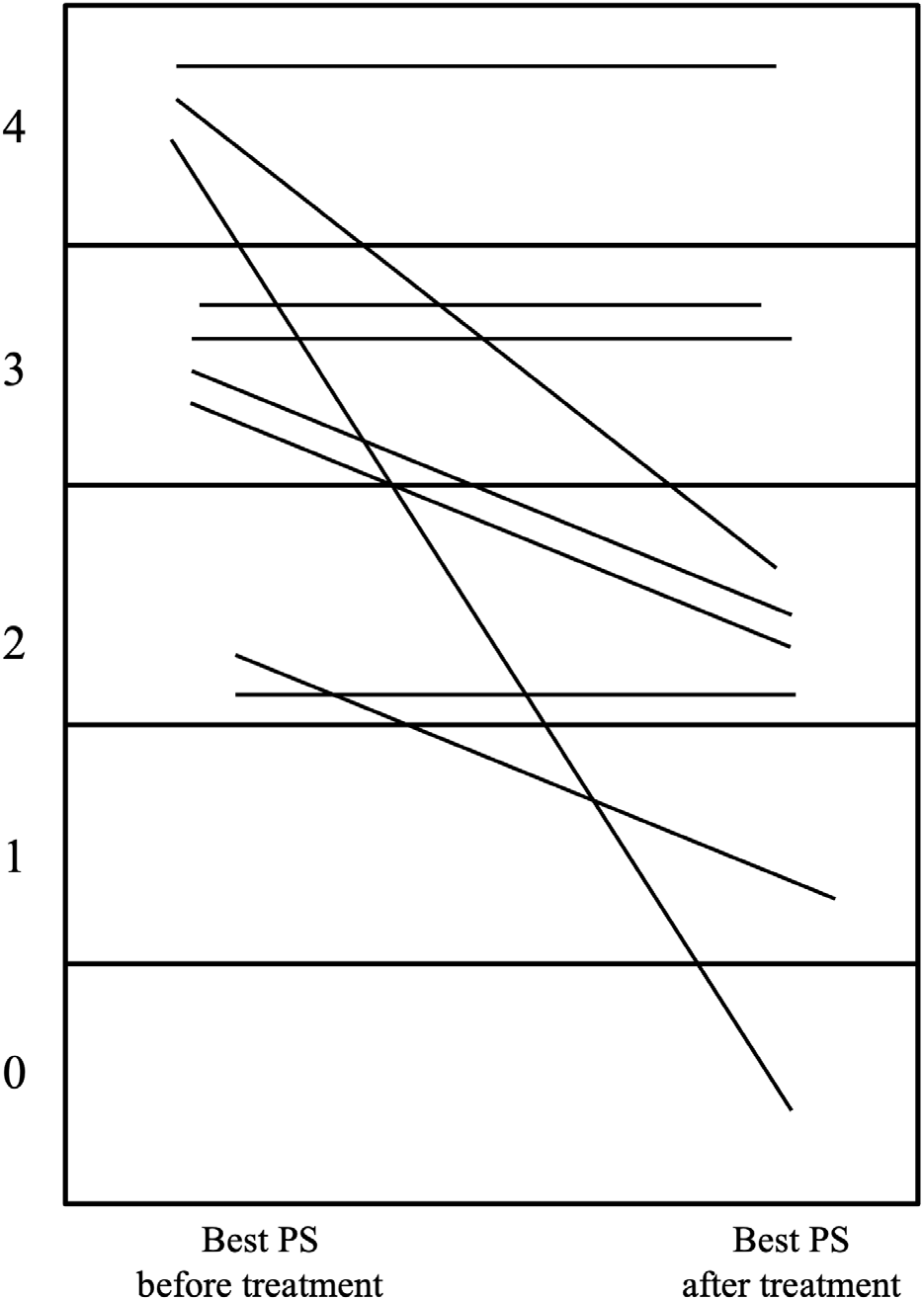
Change in performance status (PS) for each patient during treatment. Each line indicates the change in PS in patients from baseline to best status during treatment. Improvement in 56% of patients was observed.

**FIGURE 3:**
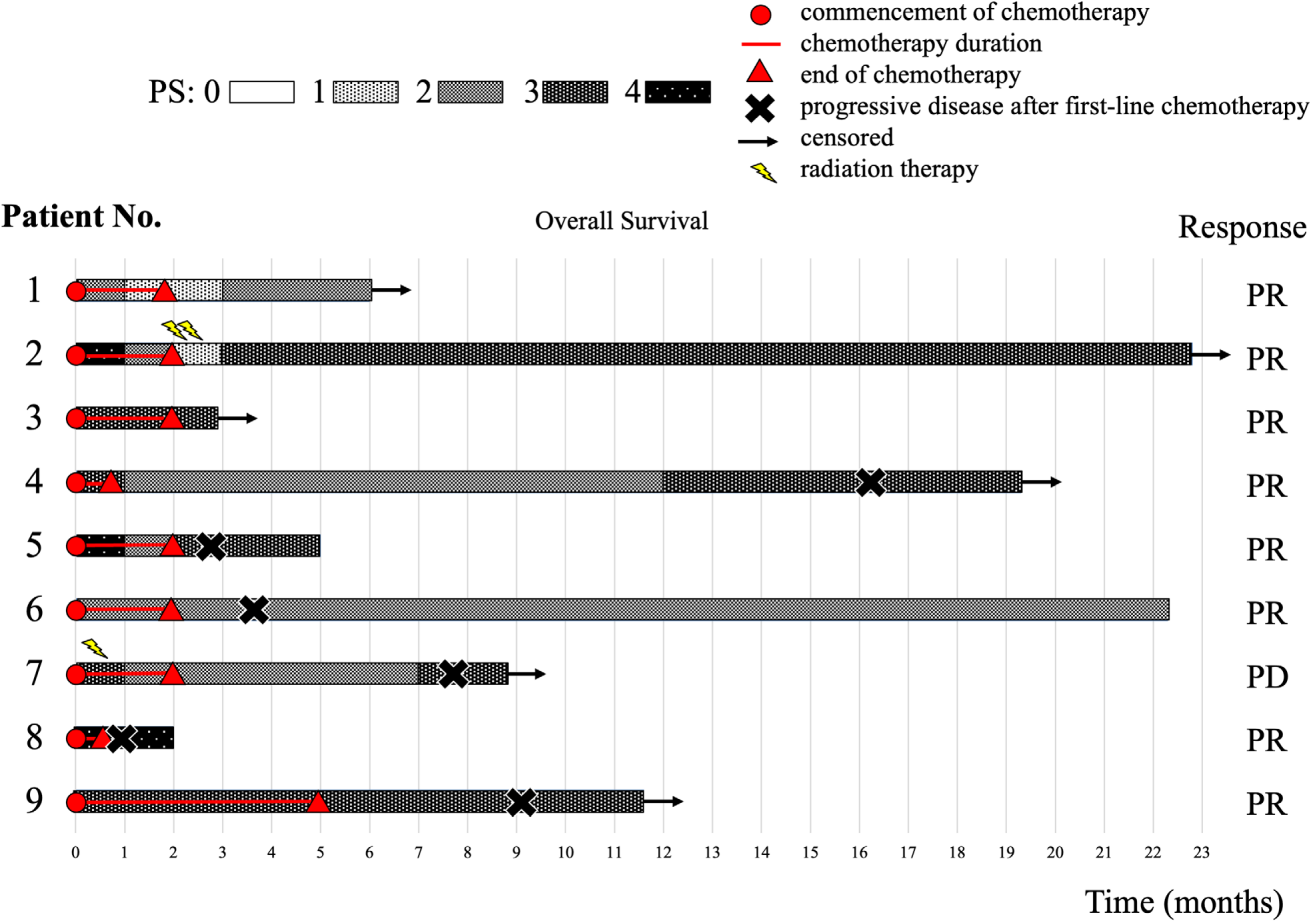
Swimmer plot of patients with paraneoplastic neurological syndrome (PNS).

Figure 2 shows the changes in PS after therapy initiation. Improvement in PS (from 3 [IQR: 2.5–4] to 2 [IQR: 1.5–3]) was observed after starting treatment for lung cancer or neurological diseases. Among patients with poor PS, the PS improved in 56% (*n* = 5) of patients. Although there was no statistically significant difference, patients with improved PS were more likely to have limited disease (60% vs. 0%, *p* = 0.17). We also created a swimmer plot focusing on the PS change after commencement of chemotherapy (Figure 3). Improvement in PS was observed relatively early after the initiation of treatment.

### PFS and OS according to the presence or absence of PNS

**FIGURE 4:**
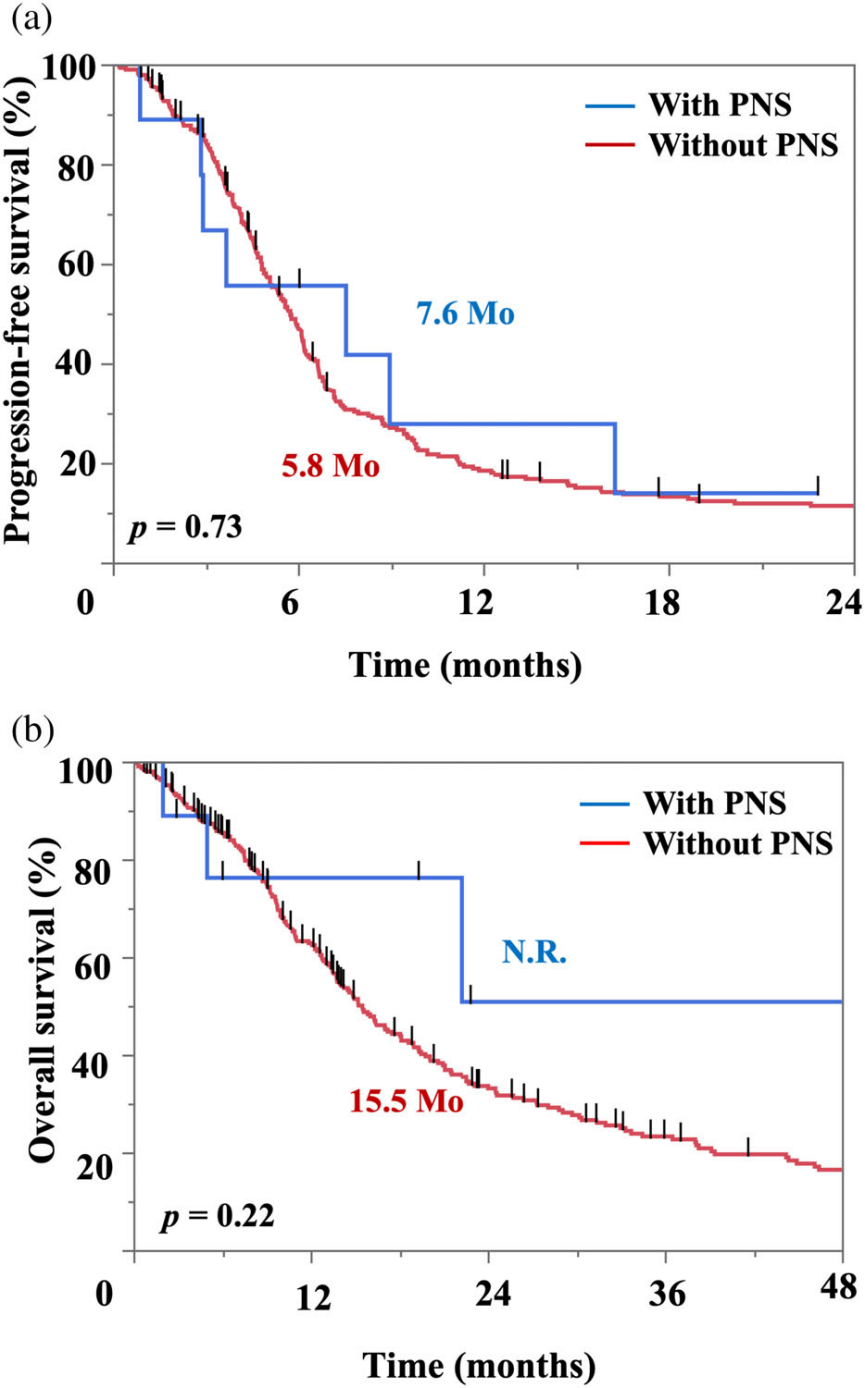
(a) Kaplan–Meier curve for progression‐free survival in patients with or without PNS. (b) Kaplan–Meier curve for overall survival in patients with or without PNS. PNS, paraneoplastic neurological syndrome.

The overall response rate was 89% in patients with PNS. The Kaplan–Meier curves for PFS and OS according to the presence or absence of PNS are shown in Figure 4a,b. Patients with PNS exhibited PFS and OS similar to those of patients without PNS (PFS: 7.6 vs. 5.7 months, *p* = 0.69; OS: not reached vs. 15.6 months, *p* = 0.23). The ORR was also similar (89% vs. 83% *p* = 1.0, shown in Table S1). Table 2 outlines the causes of death. Most of the patients were transferred to other hospitals, and the exact cause of death was known for only a few patients. One patient died because of PNS progression.

### Univariate and multivariate analyses of PFS and OS according to patient characteristics

We conducted univariate and multivariate analyses of PFS and OS according to patient characteristics, and the results are presented in Tables 3 and 4. The multivariate analysis revealed that poor PS, extended disease, and lack of RT were poor predictors of PFS, and poor PS, presence of interstitial lung disease, lack of chemotherapy, and lack of RT were poor predictors of OS, whereas the presence of PNS did not negatively predict PFS and OS.

**TABLE 3:** Cox proportional hazards model for progression‐free survival in patients with SCLC in the univariate and multivariate analyses.

Category		Univariate analysis		Multivariate analysis	
		HR (95% CI)	*p*‐value	HR (95% CI)	*p*‐value
Age	<75	Reference	0.99	Reference	0.54
	≥75	1.00 (0.77–1.31)		0.92 (0.70–1.20)	
Sex	Male	Reference	0.02	Reference	0.15
	Female	0.67 (0.48–0.93)		0.78 (0.56–1.09)	
ECOG PS	0–1	Reference	0.002	Reference	0.02
	2–4	1.57 (1.18–2.08)		1.42 (1.05–1.92)	
Presence of ILD	0–1	Reference	0.0001	Reference	0.08
	2–4	1.78 (1.32–2.40)		1.32 (0.96–1.80)	
ED or LD	LD	Reference	<0.0001	Reference	0.002
	ED	3.25 (2.41–4.37)		1.89 (1.26–2.82)	
Use of ICI	No	Reference	0.06	Reference	0.49
	Yes	1.74 (0.97–3.12)		1.24 (0.68–2.26)	
Use of RT	No	Reference	<0.0001	Reference	0.007
	Yes	0.30 (0.21–0.42)		0.52 (0.33–0.83)	
Presence of PNS	No	Reference	0.69	Reference	0.17
	Yes	0.86 (0.41–1.82)		0.57 (0.26–1.28)	

Abbreviations: CI, confidence interval; ECOG PS, Eastern Cooperative Oncology Group Performance Status; ED, extended disease; HR, hazard ratio; ICI, immune checkpoint inhibitor; ILD, interstitial lung disease; LD, limited disease; PNS, paraneoplastic neurological syndrome; RT, radiation therapy; SCLC, small‐cell lung cancer.

**TABLE 4:** Cox proportional hazards model for overall survival in patients with SCLC in the univariate and multivariate analyses.

Category		Univariate analysis		Multivariate analysis	
		HR (95% CI)	*p*‐value	HR (95% CI)	*p*‐value
Age	<75	Reference	0.01	Reference	0.25
	≥75	1.43 (1.08–1.90)		1.19 (0.88–1.60)	
Sex	Male	Reference	0.23	Reference	0.89
	Female	0.81 (0.57–1.14)		0.97 (0.68–1.39)	
ECOG PS	0–1	Reference	<0.0001	Reference	0.0002
	2–4	2.13 (1.59–2.85)		1.85 (1.34–2.56)	
Presence of ILD	0–1	Reference	<0.0001	Reference	0.0003
	2–4	2.36 (1.73–3.23)		1.83 (1.32–2.54)	
ED or LD	No	Reference	<0.0001	Reference	0.054
	Yes	2.62 (1.92–3.58)		1.49 (0.99–2.22)	
Use of chemotherapy	No	Reference	<0.0001	Reference	<0.0001
	Yes	0.05 (0.02–0.09)		0.11 (0.05–0.23)	
Use of ICI	No	Reference	0.29	Reference	0.20
	Yes	0.54 (0.17–1.70)		0.47 (0.15–1.50)	
Use of RT	No	Reference	<0.0001	Reference	0.006
	Yes	0.29 (0.19–0.43)		0.48 (0.29–0.81)	
Presence of PNS	No	Reference	0.24	Reference	0.04
	Yes	0.51 (0.16–1.58)		0.29 (0.09–0.94)	

Abbreviations: CI, confidence interval; ECOG PS, Eastern Cooperative Oncology Group Performance Status; ED, extended disease; HR, hazard ratio; ICI, immune checkpoint inhibitor; ILD, interstitial lung disease; LD, limited disease; PNS, paraneoplastic neurological syndrome; RT, radiation therapy; SCLC, small‐cell lung cancer.

## DISCUSSION

To the best of our knowledge, this is the first real‐world analysis of patients with SCLC complicated by PNS. In this study, we revealed the incidence of PNS, its characteristics, change in ECOG PS, and anticancer treatment efficacy with long‐term follow‐up data.

A study published in 1991 reported a prevalence rate of 2% for PNS in patients with SCLC,^4^ whereas a prospective study from the UK published in 2015 found a prevalence rate of 9.4% for PNS in patients with SCLC.^5^ However, differences in study design, hospital settings, and countries might have influenced the results. Our cohort included all patients diagnosed with Stages II–IV SCLC at our institute during this period. We believe that this cohort accurately reflects the community population of patients with SCLC eligible for chemotherapy or chemoradiotherapy. Our study revealed a prevalence rate of 2.8% for PNS in Japanese patients, which is consistent with the former report. With regard to the PNS phenotypes, the most prevalent was Lambert–Eaton myasthenic syndrome (44%), which was also concordant with the previous two studies.^4^, ^5^

In our cohort, all patients with PNS exhibited poor ECOG PS, mainly due to the symptoms of PNS. A notable finding of our study was that PS improvement was observed in more than half of patients after starting anticancer treatment. Improvement in PS was observed relatively early after treatment initiation. Further, treatment outcomes were comparable to those observed in the non‐PNS group, and the presence of PNS did not negatively affect PFS and OS in the multivariate analysis. As this is a real‐world based, retrospective study, anticancer treatment was administered simultaneously with various interventions such as immunomodulatory therapies and intravenous high‐dose immunoglobulin therapy, and endotracheal intubation; therefore, the precise efficacy of anticancer therapy was not evaluable. However, in the previous study, the efficacy of immunomodulatory drugs alone was limited,^8^ so we speculate that anticancer treatment had some positive effect on these patients. The favorable results shown in our study might be related to the intensive treatment other than anticancer treatment. Therefore, we speculate that PNS‐related mortality or symptoms could be reduced by rapid diagnosis and early treatment, as described in a previous study.^18^ Further studies are required to establish treatment strategies for this group of patients.

As for other treatment options, immunosuppressive therapy, such as cyclophosphamide or rituximab treatment, is one of the options. In this cohort, the most prevalent type of PNS was Lambert–Eaton myasthenic syndrome; thus, immunosuppression was not chosen. Moreover, in Japan, treatment with immunosuppressive agents is not covered by insurance; therefore, these agents are usually not chosen as treatment options. Although there are some reports on surgical treatment for patients with PNS,^12^ considering the difficulty of general anesthesia for patients with PNS, surgical intervention for recourse control is not considered a standard treatment option for patients with PNS.

Previous studies reported that programmed cell death ligand 1 inhibitors, such as atezolizumab and durvalumab, exhibited impressive efficacy in patients with SCLC.^19^, ^20^ Recently, a systematic review and population‐based study reported an increase in neurological immune‐related adverse events or exacerbation of PNS after the introduction of ICI therapy.^21^, ^22^ Because of the period of approval from the Japanese government, we included few patients who received chemotherapy and ICI therapy. Thus, there was an imbalance in the number of ICI‐treated patients between the PNS and non‐PNS groups in this study. Further studies involving patients with pre‐existing PNS who have been started on ICI therapy are warranted.

The limitation of our study includes the retrospective design from single institute; thus, the diagnostic approach and treatment protocol are not pre‐specified. However, we reviewed each clinical course according to the recent diagnostic criteria; thus, such bias was minimized in this study. Second, the sample size included in the present study is limited. Specifically, the imbalance of patient characteristics, such as sex and (eight of nine patients were male) stage (six of nine patients had extended disease) might have affected the interpretation. Preneoplastic syndrome is a heterogeneous disease; therefore, the prognosis and management strategy should be discussed according to the PNS phenotypes. In this study, we could not conduct this analysis due to the small sample size. Further large‐scale studies are required in this area.

In conclusion, this study revealed a PNS prevalence rate of 2.8%, and most patients with PNS had worse PS. However, PS improvement was commonly observed after treatment. Thus, anticancer therapies may be justified for this group of patients. In the clinical setting, physicians should recognize patients with PNS as possible candidates for anticancer therapy. Further studies on patients with pre‐existing PNS who have been initiated on ICI therapy are warranted.

## AUTHOR CONTRIBUTIONS

**Yuki Sato:** Data curation; methodology; formal analysis; funding acquisition; investigation; resources; visualization; project administration; writing—original draft; writing—review and editing. **Satoru Fujiwara:** Investigation; validation; writing—original draft; writing—review and editing. **Chigusa Shirakawa:** Investigation; resources; writing—original draft; writing—review and editing. **Ryosuke Hirabayashi:** Investigation; resources; writing—review and editing. **Kazuma Nagata:** Investigation; resources; writing—review and editing. **Atsushi Nakagawa:** Investigation; resources; writing—review and editing. **Ryo Tachikawa:** Investigation; resources; writing—review and editing. **Keisuke Tomii:** Conceptualization; methodology; project administration; funding acquisition; supervision; writing—original draft; writing—review and editing.

## CONFLICT OF INTEREST STATEMENT

Dr. Sato reported receiving a speaker's bureau fee from AstraZeneca, Chugai Pharmaceutical, MSD, Ono Pharmaceutical, Novartis, Pfizer, Taiho Pharmaceutical, Nippon Kayaku, Bristol Myers Squibb, Eli Lilly, Takeda, and Kyowa Kirin outside the submitted work. Dr. Fujiwara reported receiving a speaker's bureau fee from Biogen Japan and Daiichi Sankyo outside the submitted work. Dr. Tomii reported receiving a speaker's bureau fee from Astellas, AstraZeneca, Boehringer Ingelheim, Bristol Myers Squibb, Chugai Pharmaceutical, Daiichi Sankyo, Eli Lilly, GlaxoSmithKline, Kyorin, Kyowa Hakko Kirin, MSD, Nippon Kayaku, Novartis Pharma, Pfizer, Sanofi, Shionogi, Taiho Pharmaceutical, and Teijin Pharma and has an advisory role at Eli Lilly outside the submitted work. All remaining authors declare no conflicts of interest.

## Supporting information


**Figure S1.** (a) Kaplan–Meier curve for progression‐free survival in the overall population. (b) Kaplan–Meier curve for overall survival in the overall population.

**Table S1.** Summary of response rates to SCLC treatment (excluding patients receiving the best supportive care).

## Data Availability

The data underlying this article will be shared upon reasonable request from the corresponding author.
