# Supplementary figures and images for: Paraneoplastic neurological syndrome and its impact on the treatment outcomes of small‐cell lung cancer: A single‐center retrospective analysis
Source: Thorac Cancer. 2024 Oct 19;15(34):2418–27. doi: 10.1111/1759-7714.15472 (PMC11609046; doi:10.1111/1759-7714.15472)

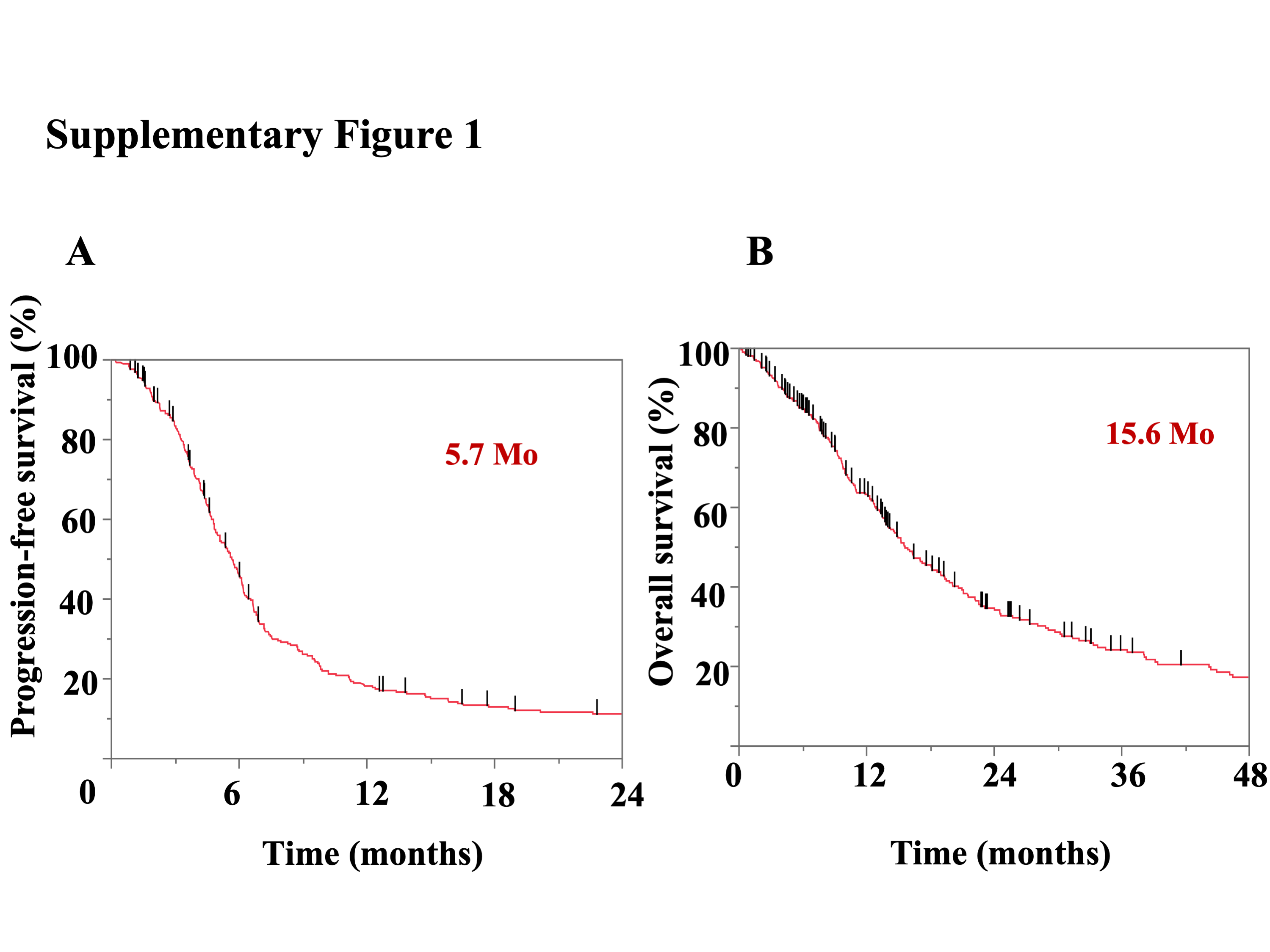

Supplement: Supplementary file 1 — Figure S1. (a) Kaplan–Meier curve for progression‐free survival in the overall population. (b) Kaplan–Meier curve for overall survival in the overall population. [file TCA-15-2418-s001.tiff]
