# Supplementary material for: Paraneoplastic neurological syndrome and its impact on the treatment outcomes of small‐cell lung cancer: A single‐center retrospective analysis
Source: Thorac Cancer. 2024 Oct 19;15(34):2418–27. doi: 10.1111/1759-7714.15472 (PMC11609046; doi:10.1111/1759-7714.15472)
Supplement: Supplementary file 2 — Table S1. Summary of response rates to SCLC treatment (excluding patients receiving the best supportive care). [file TCA-15-2418-s002.docx]

**Supplementary Table 1**. Summary of Response Rates to SCLC Treatment (Excluding Patients Receiving the Best Supportive Care)

|  | **All patients (N = 303)** | **Patients with PNS**  **(N = 9)** | **Patients without PNS**  **(N = 294)** |
| --- | --- | --- | --- |
| **Objective response (N, %)** |  |  |  |
| Complete response | 22 (7) | 0 (0) | 22 (7) |
| Partial response | 213 (70) | 8 (89) | 205 (70) |
| Stable disease | 14 (5) | 0 (0) | 14 (5) |
| Progressive disease | 32 (11) | 1 (11) | 31 (11) |
| Not evaluable | 22 (7) | 0 (0) | 22 (7) |
| **Objective response rate (%)** | 84 | 89 | 83 |
| **Disease control rate (%)** | 89 | 89 | 89 |

SCLC, small-cell lung cancer; PNS, paraneoplastic neurological syndrome.
